# Molecular adaptations specific to extreme halophilic archaea could promote high perchlorate tolerance

**DOI:** 10.1128/aem.00512-25

**Published:** 2025-05-09

**Authors:** Jorge Díaz-Rullo, José Eduardo González-Pastor

**Affiliations:** 1 Department of Molecular Evolution, Centro de Astrobiología (CAB), CSIC-INTA 120422, Madrid, Spain; 2 University of Alcalá, Polytechnic School 549696, Madrid, Spain; University of Milano-Bicocca, Milan, Italy

**Keywords:** perchlorate, extreme haloarchaea, transcriptomics, Mars exploration, molecular adaptations

## Abstract

Perchlorate is a strong chaotropic agent that causes macromolecule denaturation, DNA damage, and oxidative stress. However, perchlorate deliquescence is thought to promote the formation of liquid salt brines, even at hyper-arid and cold environments, such as the Martian regolith. For that reason, the detection of high levels of perchlorate at different locations on the Martian surface led to hypotheses about the existence of Martian microenvironments compatible with life, especially with those organisms tolerant to hyper-salinity and perchlorate. Extreme halophilic archaea have been proposed as the best candidates to inhabit those environments not only due to their high tolerance to salinity and perchlorate, but also because of their resistance to a wide variety of stress conditions. Since specific perchlorate responses remain largely unknown, in this work, we have analyzed the molecular mechanisms of perchlorate tolerance exhibited by the model extreme halophilic archaeon *Haloferax volcanii* using a transcriptomic approach. We report that perchlorate produced transcriptional effects opposite to those of salinity, and we propose that the “salt-in” strategy could promote high perchlorate tolerance in extreme halophilic archaea due to the intracellular accumulation of KCl, which may shield the chaotropic activity of perchlorate. This natural adaptation would be enhanced by changes in other stress responses like DNA repair, refolding and turnover of damaged proteins, removal of oxidative species, and tRNA modifications, among others. These results may help to understand how life could survive on Mars, now or in the past, and highlight the importance of extreme halophiles in the development of *in situ* resource utilization systems.

**IMPORTANCE:**

Perchlorate is a toxic chlorinated compound that promotes the formation of liquid salt brines, even at hyper-arid and cold environments. For the past two decades, different probes have reported high levels of perchlorate salts at multiple locations on the Martian surface, which could facilitate the presence of potentially habitable environments by specific microorganisms capable of tolerating both hyper-salinity and high perchlorate concentrations. Therefore, the significance of this research was to investigate the molecular mechanisms for perchlorate tolerance using the extreme haloarchaeon *Haloferax volcanii* as a model organism. This analysis leads to the identification of critical genes and pathways involved in perchlorate tolerance and supports that certain molecular adaptations specific to extreme haloarchaea may be responsible for the high levels of perchlorate tolerance exhibited by these microorganisms, serving as a valuable resource for Mars exploration.

## INTRODUCTION

Perchlorate (ClO_4_^−^) is a soluble anion composed of a chlorine atom surrounded by four oxygen atoms in a tetrahedral array. Perchlorate is a strong chaotropic agent and a powerful oxidant, although under most environmental conditions, it is a highly stable anion (1). Large amounts of perchlorate (0.4–0.6 wt.%) have been detected by diverse techniques at different locations of the Martian surface (2). From an astrobiological perspective, this detection has been crucial to think about the existence of certain Martian environments compatible with life (3). It is thought that perchlorate can facilitate the formation of temporary salt brines by deliquescence, a process in which hygroscopic salts absorb moisture from the atmosphere, even at hyper-arid and cold environments, such as the Martian surface (4). The formation of liquid brine droplets in the Martian regolith due to deliquescence has been proposed, which could explain the observed seasonal flows on the relatively warmer Martian slopes, called “recurring slope lineae” (5, 6). Other studies have reported the presence of large water bodies beneath the subsurface ice, which would contain perchlorate and high salinity in a similar way to the perchlorate-containing brines found beneath the Antarctic Dry Valleys, which are known to support slow-growing but active bacterial communities (2, 7). All these Martian environments would share a high salinity and perchlorate concentration mainly because most Martian liquid solutions are expected to be formed by deliquescence of perchlorate and other salts. This phenomenon would raise salt concentration, which could reach saturation (2). Therefore, high tolerance for both conditions would be a critical feature of any microorganism postulated as a candidate to inhabit the Martian surface, and halotolerant and high perchlorate-resistant organisms may fulfil such conditions to survive in Martian brines.

The main problem with perchlorate and its compatibility with life is its toxicity. Perchlorate is one of the strongest chaotropic agents according to the Hofmeister scale (8). When dissolved in water, these compounds disrupt the hydrogen bonding network between the molecules of water that form the solvation shell around macromolecules, promoting denaturation of proteins and nucleic acids, destabilization of lipid bilayers, and oxidative stress as a result of that macromolecule damage (1, 8, 9). Despite the toxicity of this compound, certain species are known to exhibit some strategies to tolerate perchlorate or even to use it as a final electron acceptor during anaerobic respiration for releasing energy. Multiple species from the three domains of life have been reported to tolerate perchlorate, especially certain cyanobacteria (up to ~0.1 M NaClO_4_), some halotolerant fungi and bacteria (up to 2.5 M NaClO_4_), and extreme halophilic archaea (0.5–1 M NaClO_4_) (2, 10–14). The molecular mechanisms of perchlorate tolerance in these microorganisms are not very well characterized to date. The halotolerant yeast *Debaryomyces hansenii* has been reported to exhibit similar stress responses to salinity and perchlorate salts, with the induction of the biosynthesis of compatible solutes, such as glycerol, that prevent chaotropic toxicity of perchlorate (15). In addition, certain proteobacteria, such as *Dechloromonas* sp. or *Azospira* sp., are dissimilatory perchlorate-reducing bacteria, which are capable of performing anaerobic respiration by reducing perchlorate or chlorate into chlorite and transforming it into Cl^−^ and molecular oxygen through the expression of perchlorate reductase and chlorite dismutase enzymes, respectively (16). Furthermore, some extreme halophilic and hyperthermophilic archaea exhibit non-canonical forms of perchlorate reduction, such as the symbiotic perchlorate reduction, in which two microorganisms collaborate to reduce perchlorate and transform it into Cl^−^ and molecular oxygen, and the cryptic perchlorate reduction, in which nitrate reductases or certain dimethylsulfoxide reductases would perform perchlorate reduction (17, 18).

Extreme halophilic archaea have been proposed as the best candidates to grow or survive on Mars not only because of their high tolerance to salinity and perchlorate, but also because they attend to other critical properties necessary to survive on Mars: resistance to high UV radiation, ionizing radiation, low temperatures, desiccation and high vacuum, anaerobic growth on perchlorate, nitrate and other electron acceptors for anaerobic respiration, and extreme longevity inside crystal salts (19). Other candidates proposed in the literature are the halotolerant yeast *D. hansenii,* which tolerates hyper-salinity and the highest perchlorate concentrations reported to date but does not resist other types of stresses present on Mars, apart from the aerobic metabolism (15), and the cyanobacterium *Chroococcidiopsis* CCMEE 029, which resists long-term desiccation, UV radiation, and ionizing radiation but shows a resistance to perchlorate that is not very high (14). For these reasons, and considering that the specific molecular mechanisms responsible for perchlorate resistance are not very well understood, a transcriptomic approach was performed to study the molecular adaptations exhibited by the model extreme halophilic archaeon *Haloferax volcanii* in response to perchlorate.

## RESULTS AND DISCUSSION

### Perchlorate may simulate the effects of decreasing salinity in *H. volcanii*

To study the molecular mechanisms triggered by *H. volcanii* in response to perchlorate, this extreme halophilic archaeon was cultured under standard conditions (Hv-YPC medium control) until exponential phase (OD_600_ = 1.0) and exposed or not to NaClO_4_ (350 mM), a concentration at which *H. volcanii* still showed an active growth (Fig. S1).

To distinguish between the effect of perchlorate anion and the increase in salinity due to the addition of the perchlorate salt, *H. volcanii* was also cultured in YPC medium supplemented with 350 mM NaCl (saline control). Total RNA was isolated and sequenced to identify the differentially expressed genes in the multiple conditions. The comparison between NaClO_4_ exposure and saline control was used to analyze the effects of perchlorate anion on the transcriptome of *H. volcanii*. The comparison between saline control and YPC medium control was studied to address the transcriptional effects of increasing salinity, and the comparison between NaClO_4_ exposure and YPC medium control was used to decipher the effects of this perchlorate salt, which should combine the effects of perchlorate anion and increasing salinity.

**Fig 1:**
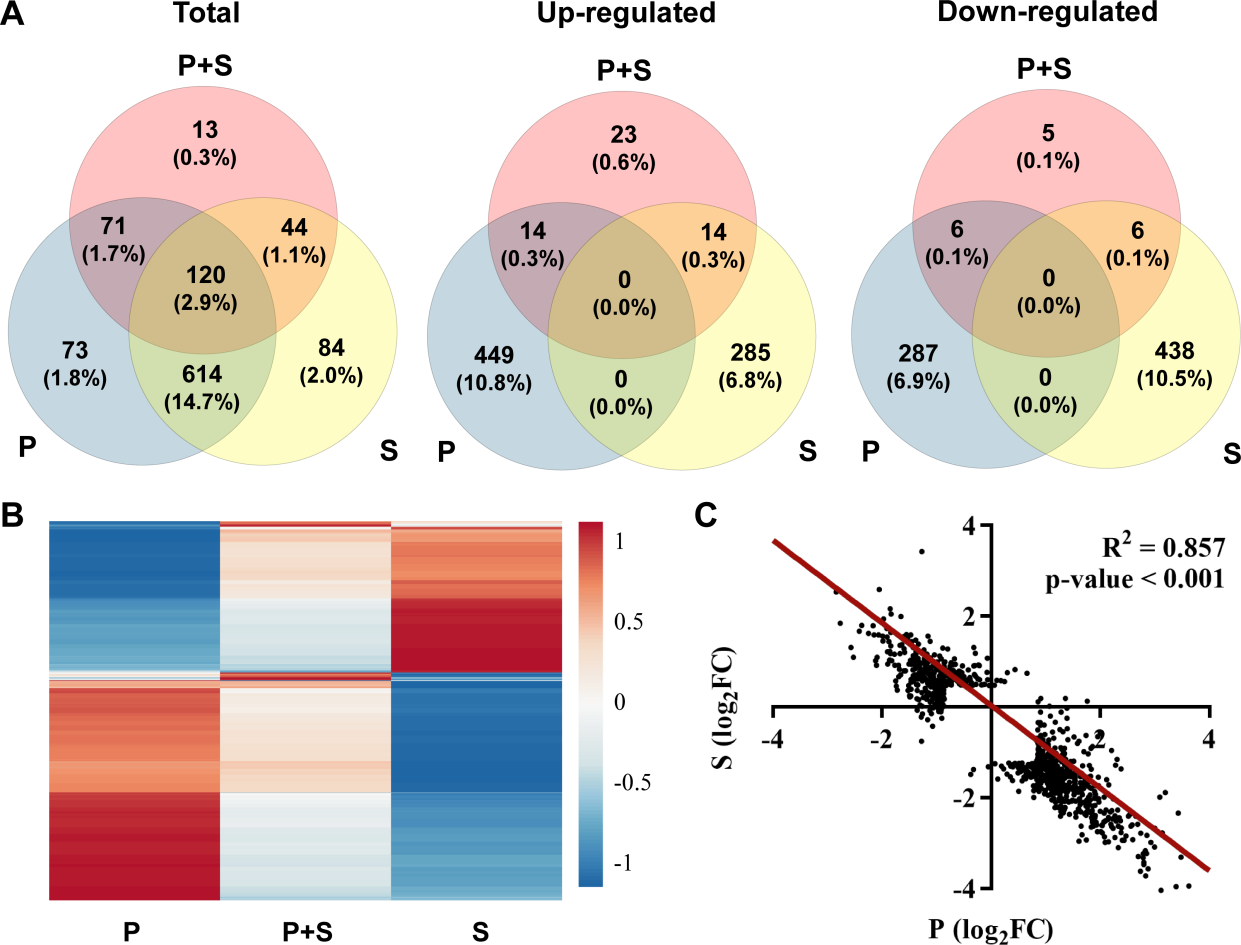
Perchlorate may simulate the effects of decreasing salinity in *H. volcanii*. (A) Venn diagrams representing the number of total, upregulated, or downregulated DEGs, together with their frequencies in the following comparisons: sodium perchlorate versus saline control (perchlorate anion effects; P), saline control versus YPC medium control (salinity effects, S), sodium perchlorate versus YPC medium control (perchlorate anion and increased salinity effects, P + S). (B) Heatmap representation of the expression changes (fold changes) of genes showing a differential expression in at least one of the three conditions (P, P + S, or S). (C) Graphical representation and linear negative regression (red line) of the log_2_-transformed fold changes (FC) of DEGs in P and S comparisons. *R*^2^: correlation coefficient.

Analysis of transcriptomic data from a global perspective showed that the expression of 790 genes (18.9%) was significantly affected by perchlorate, 743 genes (17.8%) by salinity, and only 102 genes (2.4%) by perchlorate salt (Fig. 1A; Data S1). Interestingly, we report a high overlap between the genes affected by perchlorate or salinity, although each condition caused opposite effects on the expression of these DEGs (Fig. 1A and B). In fact, we observed a linear negative correlation between the changes in gene expression due to perchlorate anion and salinity (Fig. 1C; Data S1). Therefore, the perchlorate anion may simulate the transcriptomic effects produced by a decrease in salinity. We hypothesize that this phenomenon might be related to possible opposite effects in terms of protein stability of the perchlorate anion and the high intracellular concentration of KCl present in extreme halophiles. These organisms use the “salt-in” strategy, which consists of the intracellular accumulation of KCl to maintain the osmotic balance with the hypersaline environment. Halophilic proteins show specific adaptations in their amino acid composition to be able to fold correctly under high KCl intracellular concentrations (2–5 M) (20). The most characteristic adaptation is the abundance of negatively charged amino acids, especially aspartate, present on the surface of the proteins (21). These amino acids establish weak interactions with K^+^ and water molecules that favor the folded state but destabilize the unfolded state due to electrostatic repulsion between their negative charges. In addition, interactions between K^+^ and water molecules favor the formation of a well-ordered hydration shell around the protein, even under the low water activities present in the intracellular medium of extreme halophilic microorganisms (22). These properties are characteristic of compounds with an anti-chaotropic activity: the kosmotropes. Therefore, K^+^ would act as a kosmotrope for halophilic proteins, favoring its native conformation. On the contrary, perchlorate anion, as a strong chaotrope, would destabilize that protein hydration shell favored by K^+^, inducing protein denaturation (8). Thus, we suggest that the high intracellular kosmotropic concentrations of K^+^ present in extreme halophile archaea may shield the chaotropic activity of perchlorate, such that high perchlorate concentrations would be required to produce protein denaturation and other chaotropic effects in extreme halophiles. This hypothesis is supported by the study of Carré et al., who observed that the tolerance of *H. volcanii* to chaotropic salts arises from the primary amino acid structure of its proteins that allow them to keep their folded state under high salinity conditions (23). We propose that this could be the reason behind the observed opposite transcriptional effects of perchlorate and salinity and the reported high perchlorate tolerance of extreme halophile archaea (13). In contrast to the opposite responses to salinity and perchlorate shown by *H. volcanii*, salinity and perchlorate-induced stresses share many common features in the yeast *D. hansenii*, the most perchlorate-resistant microorganism described to date (15). Some of those common responses are typical of osmotic stress, such as a high production of osmolytes like glycerol, elevated energy metabolism, or ion transport (15). In fact, several environmental halotolerant isolates have been reported to grow in the presence of significant perchlorate levels (10, 11). In the case of *H. volcanii* and probably other extreme halophilic archaea, their fundamental adaptations in terms of high KCl intracellular concentration and amino acid sequence of its proteins would be crucial for their perchlorate tolerance, although the induction of specific stress responses to perchlorate described in the following paragraphs could support robust growth under these conditions.

### *H. volcanii* exhibits a wide transcriptional adaptation toward perchlorate

**Fig 2:**
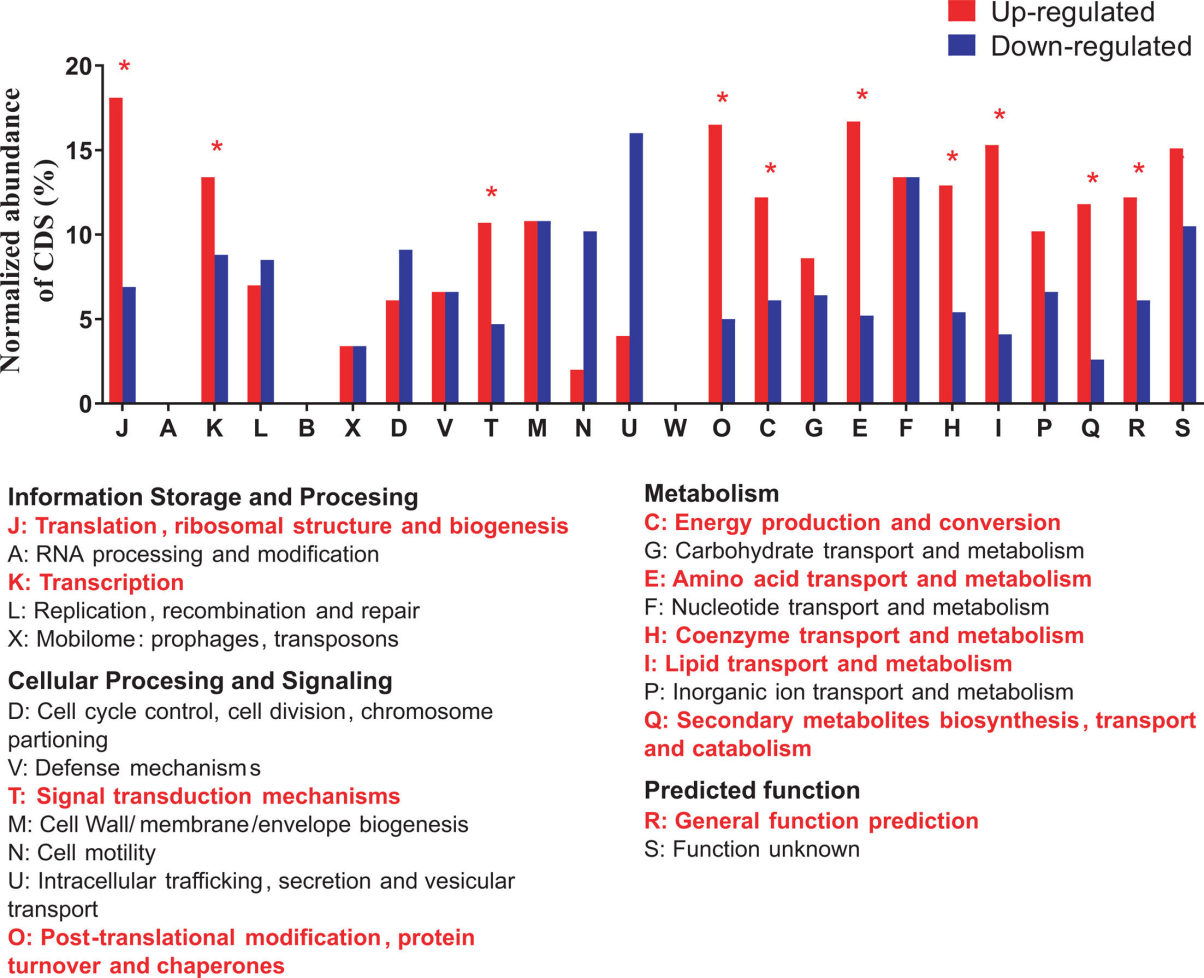
Distribution of DEGs by the effects of perchlorate anion according to COG categories. Percentages represent the number of DEGs up- (red) or downregulated (blue) relative to the respective total number of coding sequences (CDS) that belong to each COG category. Meanings of COG category abbreviations are shown below. COG categories with a significant percentage of upregulated DEGs are marked with asterisks, and their meanings are highlighted in bold red.

To analyze the cellular processes especially affected by perchlorate, the DEGs in the presence of this anion were classified according to the Cluster of Orthologous (COG) categories (Fig. 2; Data S1). In most of the categories, more DEGs were induced than repressed, such as “Translation, ribosomal structure and biogenesis (J),” “Transcription (K),” “Signal transduction mechanisms (T),” “Post-translational modification, protein turnover and chaperones (O),” “Energy production and conversion (C),” “Amino acid transport and metabolism (E),” “Coenzyme transport and metabolism (H),” “Lipid transport and metabolism (I),” or “Secondary metabolites biosynthesis, transport and catabolism (Q)” (Fig. 2). The fact that DEGs from a wide variety of processes are mostly induced may suggest that *H. volcanii* triggers a high transcriptional plasticity to adapt itself to the toxic effects of perchlorate, which may represent another explanation for the high perchlorate tolerance exhibited by this archaeon.

### Perchlorate could induce a switch toward anaerobic metabolism and removal of oxidative species in *H. volcanii*

**Fig 3:**
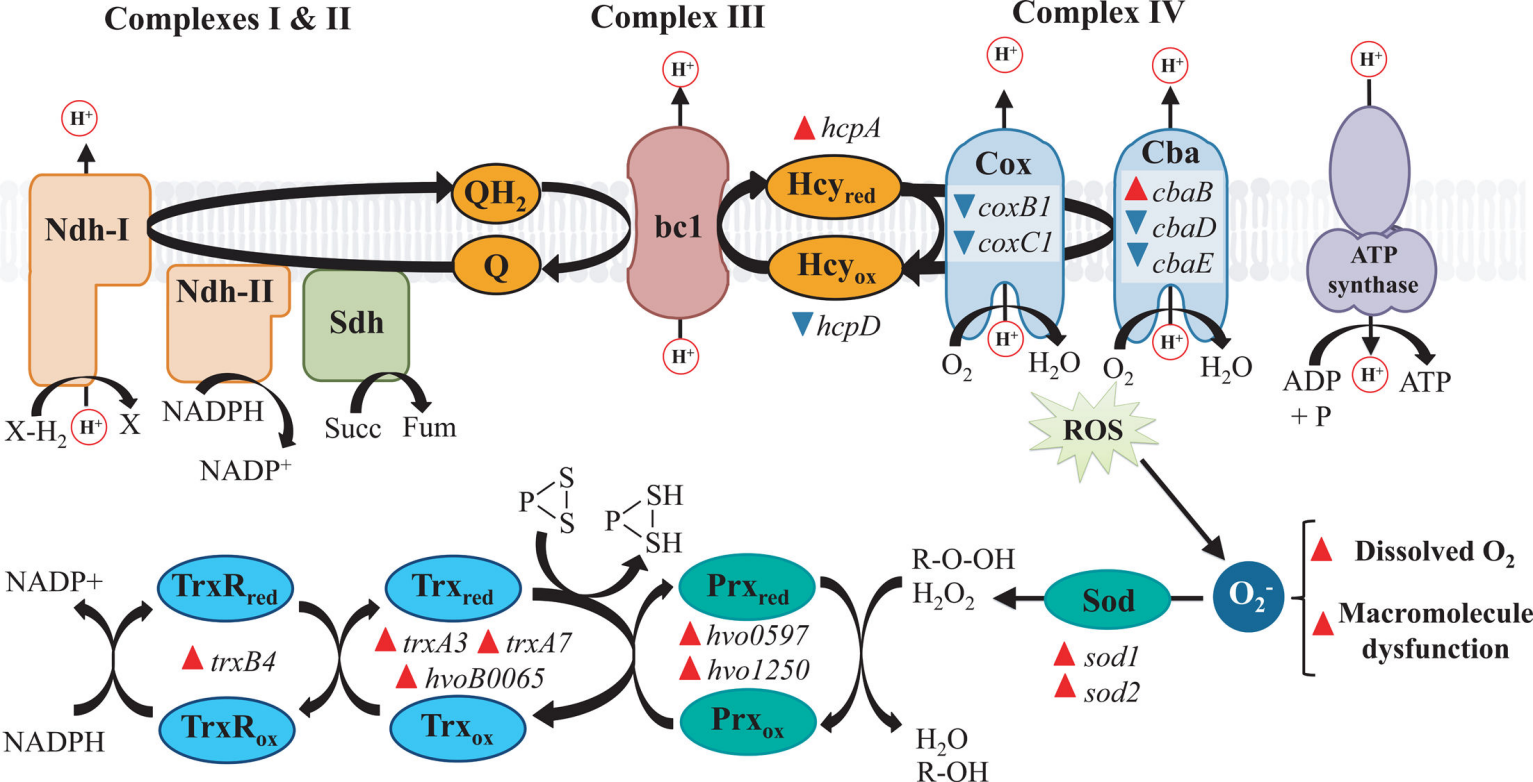
Perchlorate affects the expression of genes involved in ETC, oxidative phosphorylation, and removal of oxidative species. Gene names of DEGs belonging to those processes are shown. Expression changes of DEGs are indicated with red (upregulated) or blue (downregulated) triangles next to gene names. NDH: NAD(P)H dehydrogenase; Sdh: succinate dehydrogenase; Q: quinone; bc1: cytochrome bc1 complex; Hcy: halocyanine; Cox: cytochrome c oxidase; Cba: ba3 cytochrome c oxidase; ROS: reactive oxidative species; Sod: superoxide dismutase; Prx: peroxiredoxin; Trx: thioredoxin.

Transcriptional data indicated that the expression of multiple genes related to electron transport chain (ETC) could be altered in the presence of perchlorate (Fig. 3; Data S1). First, we observed an inhibition of several complex IV subunits Cox and Cba. Complex IV works as the final acceptor of the electrons that flow in the ETC, reducing O_2_ into water. This reaction is not always fully completed, leading to the production of reactive oxidative species (ROS) (24). For this reason, complex IV is considered one of the main sources of ROS in a cell (25). As perchlorate produces oxidative stress due to the role of its chaotropic activity in macromolecule denaturation, impaired complex IV activity may be beneficial for perchlorate tolerance. In addition, halocyanines are other proteins related to ETC that may be differentially expressed by perchlorate (Fig. 3; Data S1). These electron carriers are present in haloarchaea, transferring electrons from complex III to complex IV. There are eight different halocyanines classified into two groups and vary in their tendency to transfer electrons to complex IV. Gomez *et al.* (26) reported that mutations in different halocyanins improved the tolerance of *H. volcanii* to hypochlorite probably through a cellular shift toward expression of halocyanins less prone to transfer electrons and preventing the formation of oxygen radicals (26), and we observed a differential expression in *hcpA* and *hcpD* (Data S1). Thus, we hypothesized that differential expression of certain halocyanines and repression of Cox and Cba subunits may prevent complex IV activity and ROS formation, promoting a shift toward a more anaerobic metabolism and perchlorate tolerance. In this regard, we observed that lactate (*HVO_0214*) and alanine dehydrogenases (*HVO_2879*) were upregulated, and lactate importer (*HVO_1696*) was downregulated in perchlorate conditions (Data S1), which may support the hypothesis of a higher anaerobic metabolism in the presence of perchlorate, although it is known that *H. volcanii* can also produce lactate under certain non-anaerobic conditions (27).

On the contrary, we explained above that the strong chaotropic activity of perchlorate is known to promote the denaturation of proteins and other macromolecules, which can lead to the malfunctioning of certain processes and ROS production. The proposed decrease in natural ROS production at complex IV may help to deal with perchlorate toxicity. Additionally, it was observed an increase in the expression of genes encoding for superoxide dismutases (Sod) and peroxiredoxins Prx1 and OsmC, which will help to protect macromolecules from oxidation by ROS (Fig. 3) (28, 29). Furthermore, expression data showed an induction of genes encoding for ferredoxins and thioredoxins (Fig. 3). These proteins are maintained in a reduced state to catalyze the reduction of oxidized compounds, such as carboxylic acids, aldehydes, and alcohols. In addition, thioredoxins reduce disulfide bridges to promote the correct folding of damaged proteins (30). Furthermore, a gene encoding for a thioredoxin reductase (TrxR) was also induced with perchlorate. TrxRs play an important role in maintaining the reducing power of thioredoxins. Therefore, in addition to the observed transcriptional changes to reduce natural ROS production, such as the complex IV repression and the switch toward a more anaerobic metabolism, *H. volcanii* would increase the expression of several genes encoding for enzymes that catalyze redox reactions to degrade ROS and other oxidized compounds produced by perchlorate. We cannot discard that these adaptations of *H. volcanii* may be caused by changes in dissolved O_2_ concentration due to the increase in salinity and perchlorate anion. It is widely known that dissolved O_2_ levels are impaired by increasing salt concentration. In fact, O_2_ concentration in hypersaline environments is dramatically reduced (31). On the contrary, chaotropic salts are known to increase the solubility in water of O_2_ and other non-polar molecules (32). Therefore, an increased exposure to oxygen in the presence of perchlorate may cause an enhanced ROS production, the trigger of ROS removal mechanisms, and the switch towards a more anaerobic metabolism.

### DNA repair systems may be induced in the presence of perchlorate in *H. volcanii*

**Fig 4:**
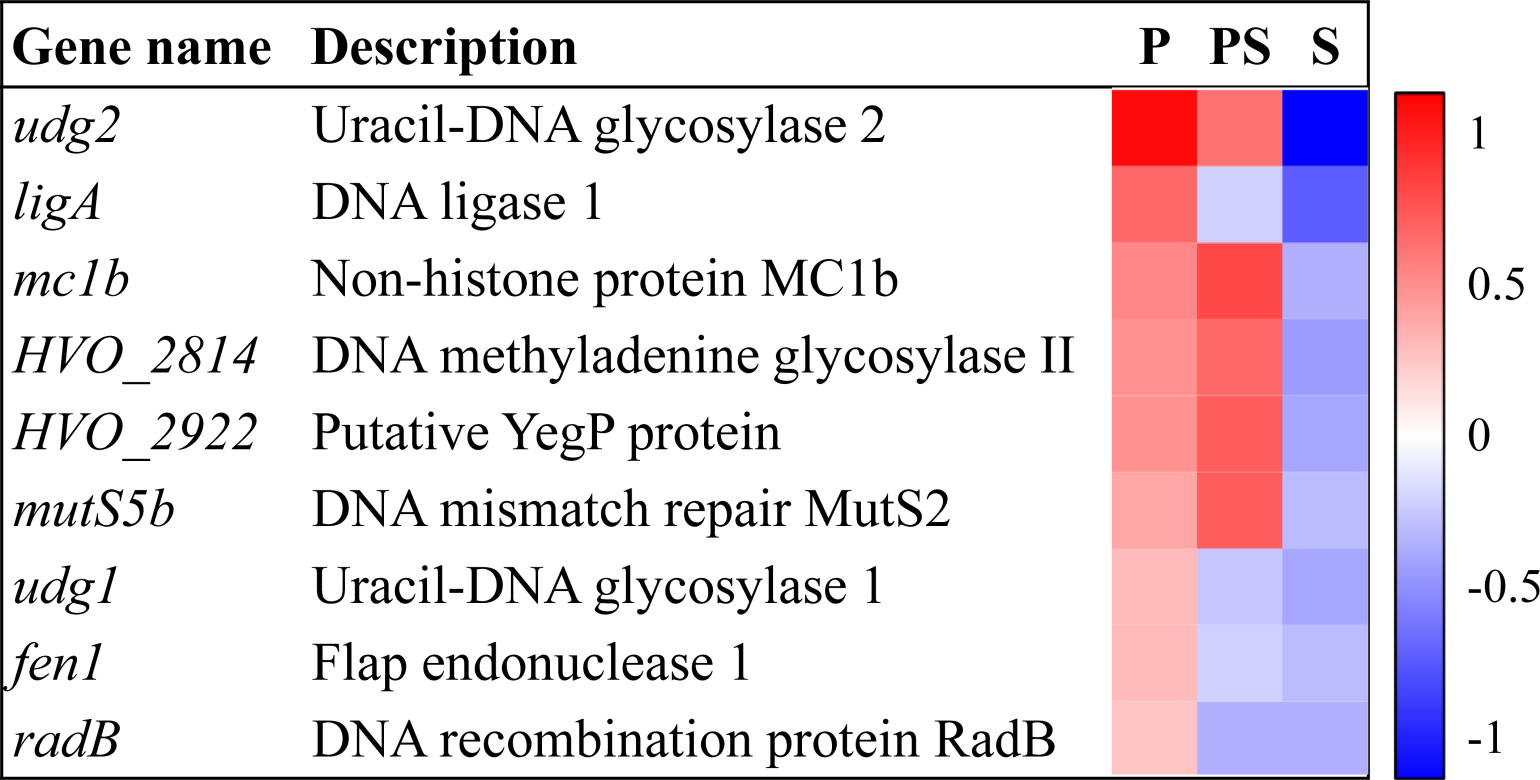
DEGs in the presence of perchlorate anion related to DNA repair systems. Heatmap represents row-normalized fold changes in the three conditions analyzed in this work (P: perchlorate anion; PS: perchlorate anion and salinity; S: salinity).

An induction of genes involved in different DNA repair systems was observed when *H. volcanii* was exposed to perchlorate (Fig. 4; Data S1). Several genes encoding proteins related to the Base Excision Repair system were up-regulated, such as uracil-DNA glycosylases (*ugd1*, *ugd2*), DNA ligase 1 (*ligA*), DNA-3-methyladenine glycosylase II family protein (*HVO_2814*), or Flap endonuclease 1 (*fen1*), or related to recombination, such as DNA repair and recombination protein RadB (*radB*), putative YegP protein (*HVO_2922*), DNA mismatch repair protein MutS2 (*mutS5b*), or Flap endonuclease 1 (*fen1*) (Fig. 4) (33–35). It has been reported that these proteins involved in DNA repair, like Fen1, play a crucial role in this process after DNA damage caused by oxidative stress (36). These data indicate that perchlorate produces DNA damage, which was previously observed in eukaryotic cells (37). Furthermore, the *mc1b* gene was also induced (Fig. 4). This gene encodes for a non-histone protein with a similar function as the histone-like proteins of bacteria, which help to compact and protect DNA (38). This result is supported by other studies of our group, in which the overexpression of a gene encoding for a histone-like protein HU from environmental microorganisms increased the resistance toward perchlorate and other types of stresses in *E. coli* (39, 40). Therefore, DNA repair and protection could play an important role in the tolerance of *H. volcanii* to perchlorate.

### Protein degradation and chaperones may represent another mechanism of resistance to perchlorate in *H. volcanii*

**Fig 5:**
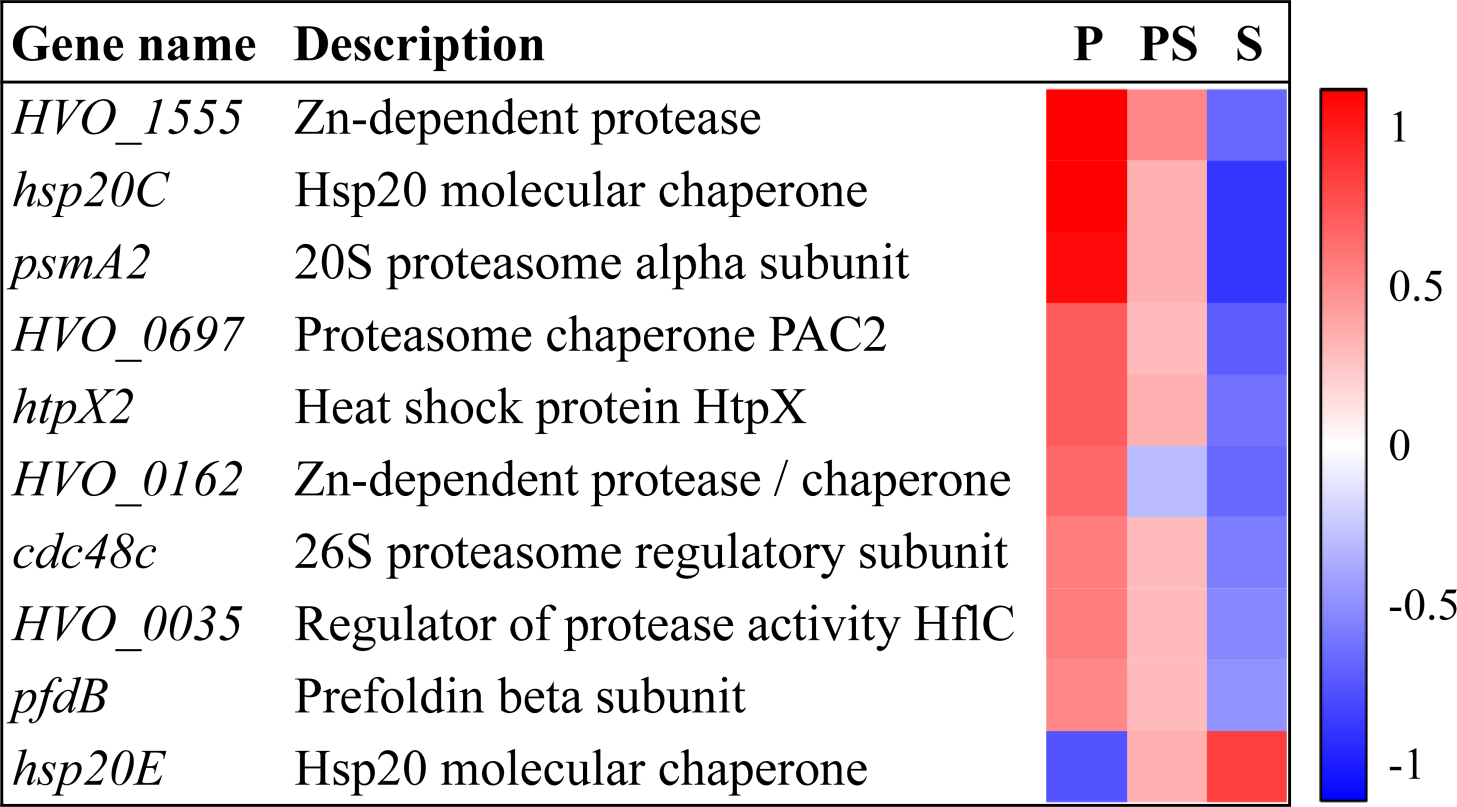
DEGs in the presence of perchlorate anion related to protein degradation and chaperones. Heatmap represents row-normalized fold changes in the three conditions analyzed in this work (P: perchlorate anion; PS: perchlorate anion and salinity; S: salinity).

One of the main effects of the chaotropic activity of perchlorate is the denaturalization of proteins. Therefore, it is reasonable to assume that protein degradation and repair of misfolded and damaged proteins may represent a crucial advantage in terms of perchlorate resistance. In fact, multiple genes belonging to the “Posttranslational modification, protein turnover, chaperones” COG category were upregulated in the presence of perchlorate (Fig. 2 and 5; Data S1). Some of those genes encoded for proteasome components (Fig. 5). The archaeal proteasome is a protein complex formed by a 20S proteolytic core particle with catalytic activity and a regulatory element with ATPase activity (41). We observed an increase in the expression of genes *psmA2* and *cdc48e* encoding for subunits of both catalytic and regulatory regions, respectively. In addition, gene *HVO_0697* was also induced with perchlorate, and this gene encodes for a PAC2 family protein, which is thought to bind immature 20S proteolytic core particles and protect the cell from an aberrant proteolytic activity of the proteasome until its assembly is fully completed (42). However, proteasome degradation is not the only way to deal with misfolded and damaged proteins. Transcriptomic data revealed that certain proteases and chaperones would be upregulated in the presence of perchlorate, such as Hsp20 (*hsp20C*), prefoldin (*pfdB*), the heat-shock protein HtpX (*htpX2*), Zn-dependent proteases (*HVO_1555*, *HVO_0162*), or the regulator of protease activity HflC (*HVO_0035*) (Fig. 5). Proteases and chaperones may help to degrade and refold those proteins damaged or misfolded by the chaotropic activity of perchlorate, respectively. In addition, these data are also supported by previous studies of our group, in which the overexpression of a gene encoding for ClpP proteases from environmental microorganisms increased the resistance toward perchlorate and other kinds of stress in *E. coli* (39, 40, 43). Clp proteases are involved in the degradation of misfolded proteins, the regulation of short-lived proteins, and the removal of dysfunctional proteins (44). Altogether, increased turnover of damaged proteins by proteasome and proteases and refolding of misfolded proteins by chaperones seem to be crucial for perchlorate resistance.

### Active translation may occur under perchlorate conditions in *H. volcanii*

**Fig 6:**
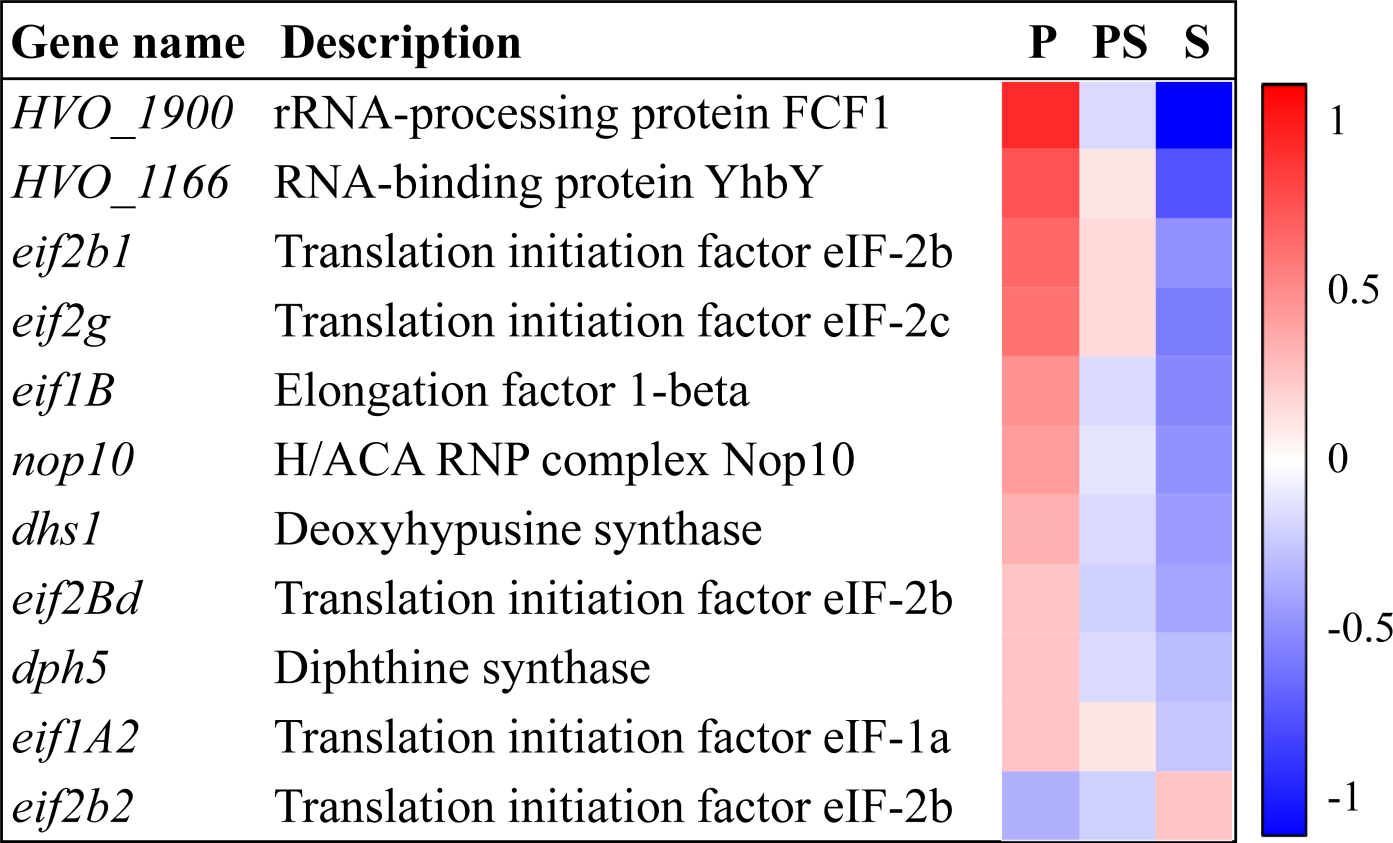
DEGs in the presence of perchlorate anion related to translation. Heatmap represents row-normalized fold changes in the three conditions analyzed in this work (P: perchlorate anion; PS: perchlorate anion and salinity; S: salinity).

Transcriptomic data revealed that *H. volcanii* may exhibit increased translational activity when exposed to perchlorate (Fig. 6; Data S1). Several genes encoding for initiation and elongation factors were upregulated with perchlorate, in addition to enzymes involved in the biosynthesis of specific modifications of these factors that ensure their correct performance, such as diphthine synthase or deoxyhypusine synthase (Fig. 6) (45). Furthermore, genes involved in the processing and maturation of ribosomes were induced, such as genes encoding for the nucleolar protein 10 (*nop10*), RNA-binding protein YhbY (*HVO_1166*), and rRNA-processing protein FCF1 (*HVO_1900*) (Fig. 6) (46–48). An active translation and ribosome biogenesis in response to perchlorate may indicate solid cell survival and growth of *H. volcanii*. This contrasts with the perchlorate response exhibited by the high perchlorate-tolerant yeast *D. hansenii*, in which ribosome biogenesis is downregulated possibly due to a slowed metabolism until stress conditions disappear.

Furthermore, numerous genes encoding for ribosomal proteins were differentially expressed in the presence of perchlorate, which suggests that the composition of the ribosomes is changing under that condition (Data S1). It was reported that the alteration of the proportions of ribosomal proteins may help cells to adapt themselves to different stress conditions, in a process called “ribo-tuning.” This process may promote a biased translation, enhancing or repressing the translation of certain mRNAs (49, 50). Therefore, changes in the ribosomal composition might regulate the translation of certain mRNAs particularly relevant during the perchlorate stress response.

### Amino acid and nucleotide metabolism of *H. volcanii* is altered in response to perchlorate

**Fig 7:**
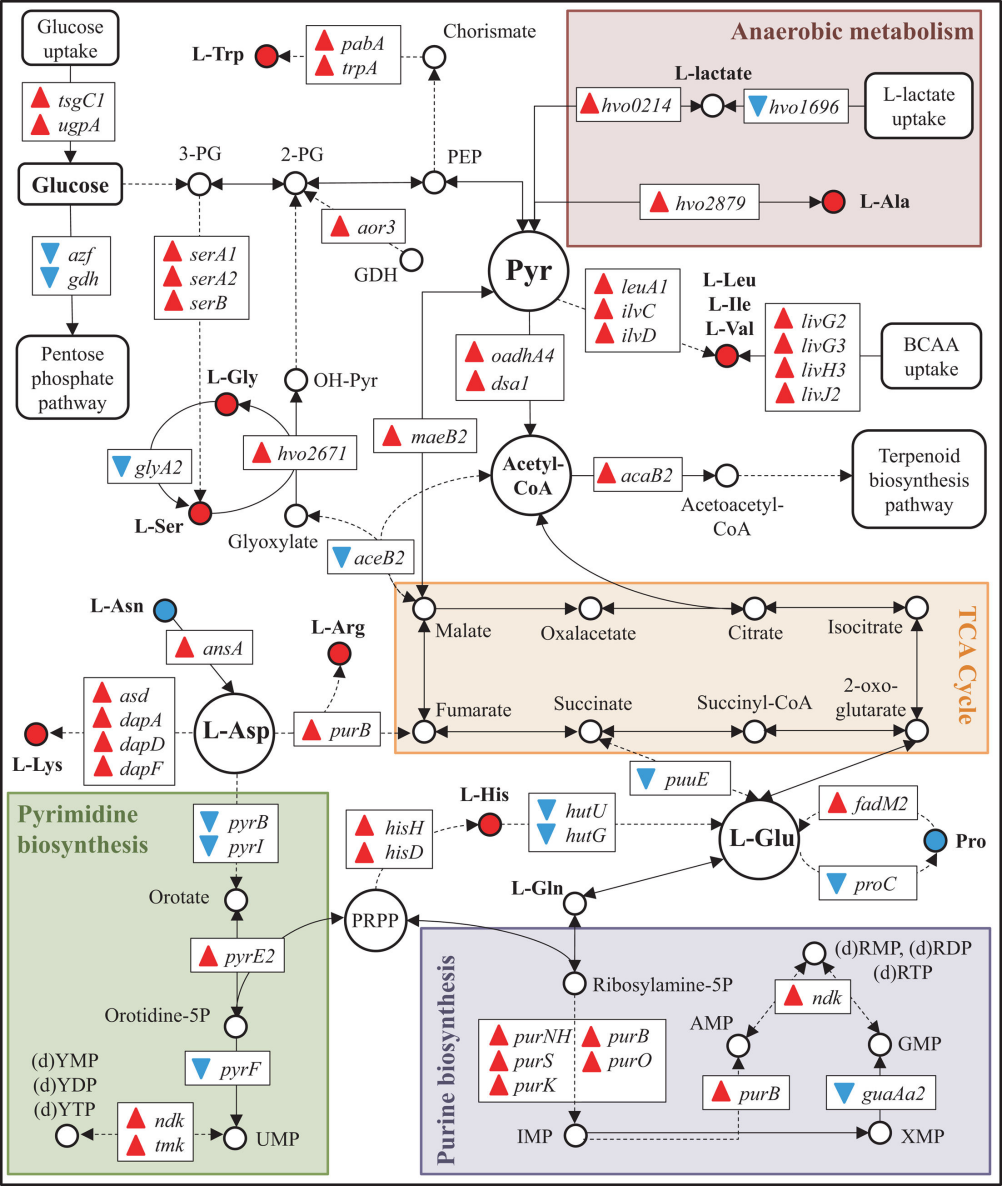
The energy, amino acid, and nucleotide metabolism of *H. volcanii* is altered in response to perchlorate. DEGs in the presence of perchlorate anion involved in those metabolic processes are shown. Triangles represent up- (red) or downregulation (blue) of DEGs. Metabolites are represented by circles, single reactions by arrows, and multiple reactions by dashed arrows. Color of circles representing amino acids represents their production/accumulation (red) or degradation/consumption (blue). PG: phosphoglycerate; PEP: phosphoenolpyruvate; GDH: glyceraldehyde; Pyr: pyruvate; PRPP: phosphoribosyl diphosphate; YMP/YDP/YTP: pyrimidine mono/di/triphosphate nucleotides; UMP: uridine monophosphate; RMP/RDP/RTP nucleotides: purine mono/di/triphosphate; AMP: adenosine monophosphate; IMP: inosine monophosphate; XMP: xanthosine monophosphate.

In the case of nucleotide metabolism, numerous genes involved in purine anabolism were upregulated in the presence of perchlorate maybe to increase the availability of AMP and ADP to produce ATP, which would be needed by chaperones and proteasome to refold or degrade damaged proteins, to maintain an active protein turnover at the translational level, remove ROS, or produce c-di-AMP required for a correct osmoregulation (Fig. 7; Data S1) (51, 52). In terms of amino acid metabolism, it is worth noting that most of the DEGs involved in this process were regulated in such a way that general amino acid anabolism seemed to be induced. This fact is followed by the upregulation of numerous genes encoding for dipeptide permease (Dpp) proteins, ATP-binding cassette (ABC) transporters that import dipeptides (Fig. 7; Data S1) (53). The hypothetical accumulation of amino acids could be related to the active translation exhibited by *H. volcanii* to adapt itself to perchlorate conditions and to increase the turnover of those proteins damaged by the chaotropic activity of perchlorate.

### tRNA archaeosine modification impairs tolerance toward perchlorate and low salinity in *H. volcanii*

**Fig 8:**
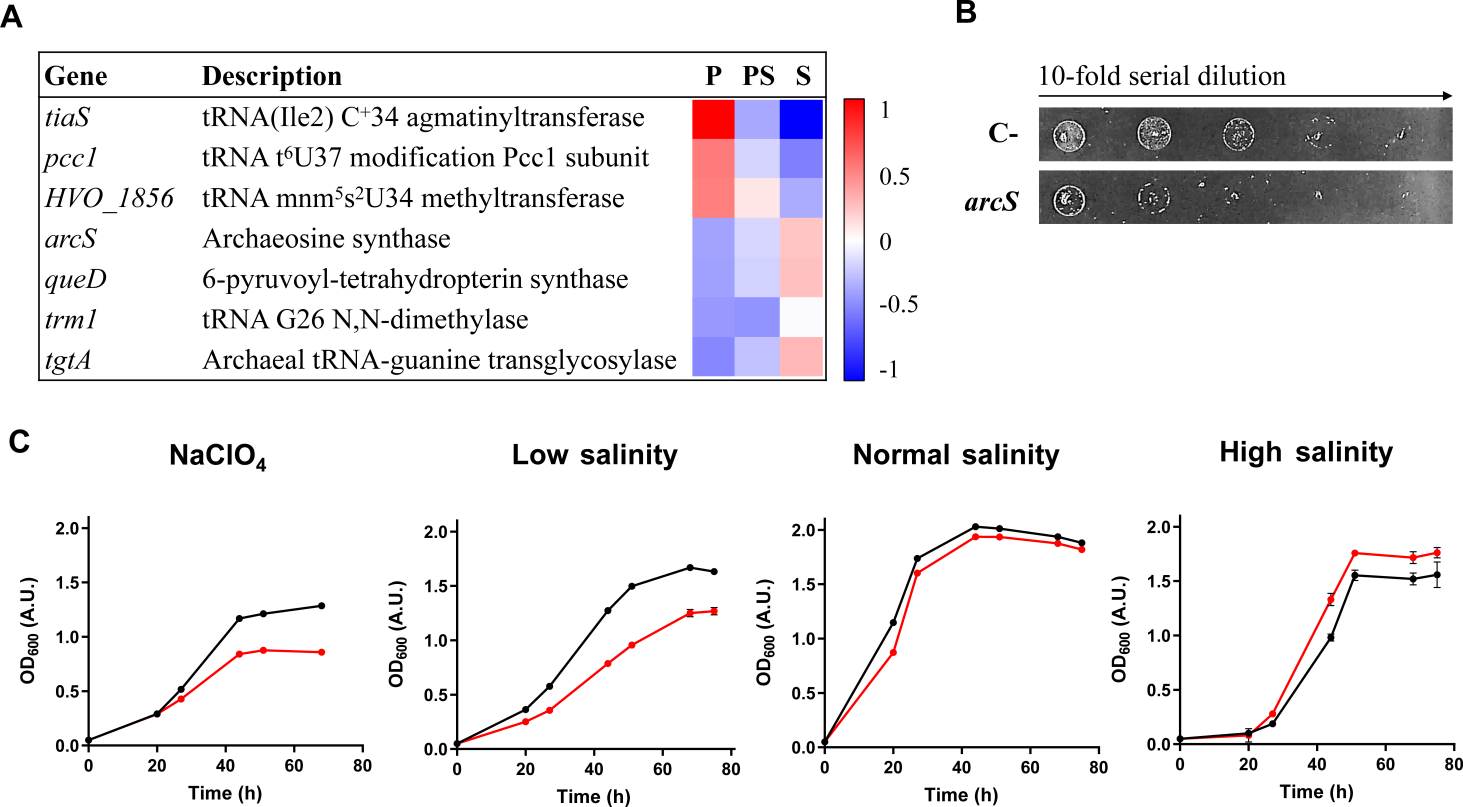
Archaeosine affects the tolerance of *H. volcanii* to perchlorate and low salinity. (A) Heatmap represents row-normalized fold changes in the three conditions analyzed in this work (P: perchlorate anion; PS: perchlorate anion and salinity; S: salinity) of DEGs involved in tRNA modifications. (B) Perchlorate resistance test of *H. volcanii* strain overexpressing *arcS* gene. Cultures of control (empty plasmid) and *arcS* strains were grown overnight at 42°C; OD_600_ was adjusted to 0.6; and 10 µL drops of 10-fold serial dilutions were plated onto Hv-YPC-NOV agar plates supplemented with 600 mM NaClO_4_. Plates were incubated at 42°C for 5 days. (C) Growth curves of *H. volcanii* (black line; control with empty plasmid) and *H. volcanii* overexpressing *arcS* gene (red line) in the presence of 350 mM NaClO_4_ and low (11% sea water), normal (18% sea water), and high (30% sea water) salinities. Data represent the mean ± standard deviation (*n* = 3).

In the presence of perchlorate, multiple genes related to the biosynthesis of certain tRNA modifications were differentially expressed, such as archaeosine (*arcS*, *tgtA*, *queD*), dimethylguanosine (*trm1*), agmatidine (*tiaS*), 5-methylaminomethyl-2-thiouridine (*hvo1856*), and N6-threonylcarbamoyladenosine (*pcc1*) (Fig. 8A; Data S1). In previous studies of our group, the expression in *E. coli* of genes involved in the biosynthesis of certain tRNA modifications increased the resistance of this bacteria toward perchlorate and other stress conditions (39, 43). Therefore, we explored whether the overexpression of some of those DEGs would alter the tolerance of *H. volcanii* to perchlorate. We observed that the overexpression of the *hvo1856* gene reduced the cell growth of *H. volcanii* in the presence of perchlorate, and *arcS* overexpression impaired its growth in the presence of both perchlorate and low salinity (Fig. 8B and C; Fig. S2; Data S1). *hvo1856* encodes for a tRNA 5-methylaminomethyl-2-thiouridine methyltransferase involved in the biosynthesis of mnm^5^s^2^ tRNA modification. It is reported in bacteria that this modification could enhance the misreading of Asn codons by tRNA-Lys (54). Therefore, the presence of mnm^5^s^2^-tRNA-Lys may increase the Lys protein content, impairing protein stability and reducing perchlorate resistance. However, *hvo1856* was upregulated with perchlorate anion; thus, further research is needed to understand the role of this modification in archaea. The *arcS* gene encodes for the enzyme archaeosine synthase, which catalyzes the last step of the archaeosine biosynthetic pathway. Archaeosine replaces guanine located at position 15 inside the D-loop of the tRNAs of almost all archaeal species (55). Its function remains elusive to date, although it was proposed that archaeosine may play a structural role through the formation of a Levitt base pair with the nucleotide 48 of the tRNAs (56). This interaction has been experimentally verified in the hyperthermophile *Thermococcus kodakarensis* (57). Considering this information, it would be reasonable to suggest that the reduced tolerance to perchlorate of *H. volcanii* when overexpressing *arcS* is caused by structural alterations of the tRNAs. However, this statement leads to two problems. First, archaeosine is thought to increase tRNA stability, whereas perchlorate would impair that stability through its chaotropic activity. Therefore, it makes more sense that *arcS* overexpression increases perchlorate tolerance (8). Second, it is reported that the absence of archaeosine leads to a variety of phenotypes, one opposed to the other, in diverse archaea: loss of thermophily in *T. kodakarensis*, no phenotype in the mesophile *Methanosarcina mazei,* and cold sensitivity in *H. volcanii* (57, 58). These data may indicate that structural stability is not the main role of archaeosine, and other hypotheses are needed to clarify its physiological role. In a previous study of our group, it was reported that another tRNA modification, called queuosine (Q), is involved in perchlorate resistance in bacteria (59). Q is present in bacteria and eukaryotes, not in archaea, at the wobble anticodon position 34 of tRNAs harboring the anticodon sequence 5′-GUN-3′ and controls the translational efficiency of NAU codons (59–61). Archaeosine and Q share part of their biosynthetic pathway, suggesting similarities in their functions. Thus, we propose that archaeosine may exhibit in archaea a translational role similar to that of Q in bacteria and eukaryotes, which might explain the variety of archaeosine-related phenotypes. Further research will be required to verify this hypothesis about the physiological role of archaeosine in archaea.

### Conclusion

Although a wide variety of perchlorate-tolerant organisms have been reported in the literature to date, extreme halophilic archaea seem to fulfill most of the requirements needed to inhabit the putative, liquid, and perchlorate-rich brines on the Martian surface (19). For that reason, we analyzed the transcriptional responses to the presence of perchlorate of the extreme halophilic archaeon *H. volcanii*. It was observed that perchlorate anion produced opposite effects to salinity, and we propose that the high KCl intracellular concentration present in extreme halophilic archaea may behave as a kosmotrope in this context, shielding the chaotropic effects of perchlorate. In addition, *H. volcanii* showed great transcriptional plasticity in response to perchlorate: switch toward anaerobic metabolism to reduce natural ROS production, increased ROS removal, refolding and degradation of damaged proteins, active translation and amino acid production to perform the turnover of damaged proteins, and translational fine-tuning via tRNA modifications like archaeosine. Thus, the main mechanism of perchlorate tolerance of extreme halophilic archaea may result from its fundamental specific adaptations of the halophilic proteins and the high KCl intracellular concentrations derived from the “salt-in” strategy, although inducing other general stress responses like the ones reported here would support a robust growth of *H. volcanii* under these conditions. Their results may help to understand how life could survive on Mars or how putative ancient Martian life could have evolved. High salinity and perchlorate conditions seem to have been ubiquitous on Mars for hundreds of millions of years. Therefore, putative Martian ancient life may have evolved to develop strategies to survive under those conditions. In this sense, the “salt-in” strategy would represent an elegant biological solution to the problem of tolerating high levels of salinity and perchlorate. Thus, attending to our results, it would be reasonable to think about the possibility of finding present or past Martian life forms, which could exhibit a mechanism like the “salt-in” strategy. In addition to that, and due to the long durations and cost of the trips, *in situ* resource utilization represents a crucial strategy in the future exploration of Mars. The use of microorganisms for that purpose has been proposed because they would need fewer requirements and offer a wide variety of chemical solutions for producing food, fuel, and materials (14, 62, 63). Therefore, the characterization of the molecular mechanisms to perchlorate tolerance exhibited by extreme halophilic archaea highlights the use of these organisms in the development of *in situ* resource utilization strategies.

## MATERIALS AND METHODS

### Bacterial strains, media, and culture conditions

Bacterial strains used in this work were *Escherichia coli* DH10B (Invitrogen), a non-restricting and non-methylating *E. coli* JTU007 strain (64), and *H. volcanii* DS70 strain (kindly provided by Dr. Josefa Antón, Universidad de Alicante, Spain). *E. coli* DH10B and JTU007 strains were routinely grown in lysogeny broth (LB) medium (Condalab) at 37 or 30°C, respectively. *H. volcanii* DS70 strain was cultured at 42°C in Hv-YPC, a medium containing yeast extract (Biolife), peptone (Oxoid), casamino acids (BD), and 18% w/v of sea water (65). When required, antibiotic final concentrations were 100 µg ml^−1^ ampicillin (AMP) and 0.3 µg mL^−1^ novobiocin (NOV). For solid cultures, growth media were supplemented with 15 g L^−1^ agar or noble agar (Difco) in the case of Hv-YPC medium. Liquid cultures were shaken on an orbital platform operating at 200 rpm.

### RNA isolation for transcriptomics

*H. volcanii* DS70 preculture was grown at 42°C in Hv-YPC medium until an OD_600_ of 1.0 was reached (exponential phase). Cells from grown precultures were used to set three 10 m Lcultures, one for each experimental condition: control, supplementation with NaClO_4_ or with NaCl (saline control). Cultures were incubated for 30 min at 42°C in 50 mL closed tubes in order to analyze the short-term transcriptional response of *H. volcanii* to perchlorate. To select the optimal NaClO_4_ concentration and prevent exposing *H. volcanii* to lethal conditions, 10 mL cultures adjusted to an OD_600_ of 1.0 were incubated with different concentrations of NaClO_4_ (300, 350, and 400 mM) for 6 h, and OD_600_ was measured every hour. Accordingly, 350 mM of NaClO_4_ was selected, as it was the maximum concentration at which *H. volcanii* still showed active growth. After incubation, cells from 1 mL of each culture was harvested by centrifugation (5 min, 4,000 *g*).

Total RNA was extracted and purified using QIAGEN RNeasy Mini Kit (QIAGEN) according to the manufacturer’s instructions. Genomic DNA was removed by DNase digestion (QIAGEN). RNA concentrations were determined in Qubit Fluorometer 3.0 (Invitrogen) using Quant-iT RNA Assay Kit (Invitrogen). RNA integrity was measured in the Agilent 2100 Bioanalyzer (Agilent Technologies, Palo Alto, CA, USA) with the Agilent RNA 6000 Nano Kit (Agilent Technologies). RNA integrity numbers (RIN) of all the samples were higher than 8.5, indicating adequate quality for sequencing.

### RNA sequencing

Library generation and RNA sequencing were performed using 1 µg RNA for each sample at FISABIO (Valencia, Spain) following manufacturer’s instructions. rRNA was depleted from total RNA using Ribo-off rRNA Depletion Kit (Vazyme Biotech Co.) compatible with *H. volcanii* DS70. One library per sample was prepared using the TruSeq stranded mRNA Library Prep Kit (Illumina). Libraries sequencing was performed on one high-output flow cell of NextSeq 500 (Illumina). Sequencing readings were single-end with a length of 75 bp, and the estimated coverage was around 20 million reads per sample (BioProject accession number PRJNA967210).

### Data processing

Quality control of the high-throughput sequence data was performed using FastQC v0.11.9 tool, and low-quality reads were removed using Trimmomatic v0.39 (66) with standard parameters. After cleanup, reads were aligned against the *H. volcanii* DS2 reference assembled genome using Bowtie 2 v2.3.5.1 (67) and sorted and indexed with Samtools 1.9 (68). DESeq2 was used to calculate the expression levels, identify differentially expressed genes between pairs of treatments, and assign a *P*-adjusted value for statistical significance (69). We considered a *P*-adjusted value < 0.1 and a fold change cutoff of ±1.8 for selecting differentially expressed genes (DEGs) during the very short-term transcriptional response toward erchlorate exposure.

### Construction of *H. volcanii* strain overexpressing genes involved in biosynthesis of tRNA modifications

Genes from *H. volcanii* encoding for enzymes involved in the biosynthesis of several tRNA modifications (*arcS*, *hvo1856*, *tiaS*, *pcc1*, *trm1*) were cloned using specific primers (Table S1) in the pAJ plasmid, a shuttle expression vector with a promoter functioning in both *H. volcanii* and *E. coli* (70). Constructions were introduced into the *E. coli* DH10B strain by electroporation, and positive clones were selected in LB-AMP plates and verified by sequencing. To deplete methylations from the construction, plasmids isolated from positive DH10B clones were introduced into the *E. coli* JTU007 strain by electroporation. Positive JTU007 clones were selected in LB-AMP plates and verified by sequencing. Non-methylated construction isolated from positive JTU007 clones was used to transform *H. volcanii* DS70 strain following the polyethylene glycol-mediated spheroplast transformation method (71, 72). Clones were selected in Hv-YPC-NOV plates for 4 days at 42°C and verified by sequencing. It was not possible to obtain clones overexpressing the *trm1* gene.

### Growth curves for testing perchlorate and salinity tolerance

*H. volcanii* DS70 overexpressing biosynthetic genes of tRNA modifications were grown until stationary phase at 42°C in Hv-YPC-NOV medium. These cells were used to set new 15 mL cultures at an adjusted OD_600_ of 0.05 in Hv-YPC-NOV medium containing 11 (low salinity), 18 (normal salinity), or 30% (high salinity) saltwater or 18% with 350 mM NaClO_4_. Cultures were grown for 3 days at 42°C and 200 rpm in 50 mL closed tubes arranged horizontally on the incubator to optimize shaking and avoid medium evaporation and salt concentration. OD_600_ was measured at different time points.

### Perchlorate tolerance test by drop assay

A drop assay was used to determine perchlorate resistance capability of the *H. volcanii* DS70 pAJ/*arcS* strain. Cells were grown in Hv-YPC-NOV at 42°C until stationary phase. OD_600_ was adjusted to 0.6, and 10-fold serial dilutions were performed. Finally, 10 µL drops of each dilution were sequentially plated onto Hv-YPC-NOV agar plates supplemented with 600 mM NaClO_4_. Plates were incubated at 42°C for 5 days. Drops were also inoculated on Hv-YPC-NOV agar to verify that the cell viability of both strains was similar. Images were taken using the precision DP70 CCD camera (Olympus).

### Statistical methods

GraphPad Prism version 9.00 (GraphPad Software, La Jolla, CA, USA) was used to calculate statistical parameters, including the values of mean and standard deviation (SD) based on the data sets from independent experiments, and the correlation between the fold changes of DEGs in perchlorate and salinity conditions. Heatmap representation of the fold changes of all DEGs was created using the ClustVis tool, applying unit variance row scaling (73).

## Data Availability

Transcriptomic data used in this study are publicly available at the BioProject database of the NCBI (accession number PRJNA967210).

## SUPPLEMENTAL MATERIAL

Data Set S1 aem.00512-25-s0001.xlsx Transcriptomic data.

Supplemental figures and tables aem.00512-25-s0002.pdf Figures S1 and S2; Table S1.

ASM does not own the copyrights to Supplemental Material that may be linked to, or accessed through, an article. The authors have granted ASM a non-exclusive, world-wide license to publish the Supplemental Material files. Please contact the corresponding author directly for reuse.
