# Supplementary material for: Molecular adaptations specific to extreme halophilic archaea could promote high perchlorate tolerance
Source: Appl Environ Microbiol. 2025 May 9;91(6):e00512-25. doi: 10.1128/aem.00512-25 (PMC12175516; doi:10.1128/aem.00512-25)
Supplement: Supplemental figures and tables — Figures S1 and S2; Table S1. [file aem.00512-25-s0002.pdf]

Supplemental Material for: Molecular adaptations specific to extreme halophilic archaea could promote high perchlorate tolerance

Jorge Díaz-Rullo[a,b]# and José Eduardo González-Pastor[a,]†

[a]Department of Molecular Evolution, Centro de Astrobiología (CAB), CSIC-INTA, Carretera de Ajalvir km 4, Torrejón de Ardoz, 28850, Madrid, Spain.

[b]University of Alcalá, Polytechnic School, Ctra. Madrid-Barcelona, Km. 33.600, 28871, Alcalá de Henares, Madrid, Spain.

Running Title: Perchlorate tolerance mechanisms in extreme haloarchaea

#Address correspondence to Jorge Díaz-Rullo: jdiaz@cab.inta-csic.es, drajesp@gmail.com.

†Deceased.

SUPPLEMENTARY FIGURES AND TABLES

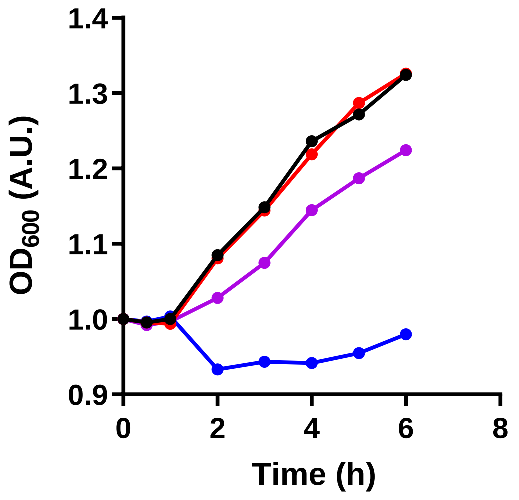

Figure S1. Growth of *H. volcanii* in the presence of different $NaClO_4$ concentrations. *H. volcanii* was grown in YPC medium until exponential phase (OD_{600} = 1.0), when was exposed to different $NaClO_4$ concentrations (black: control; red: 300 mM; purple: 350 mM; blue: 400 mM). Data represent the mean ± S.D. (n=3).

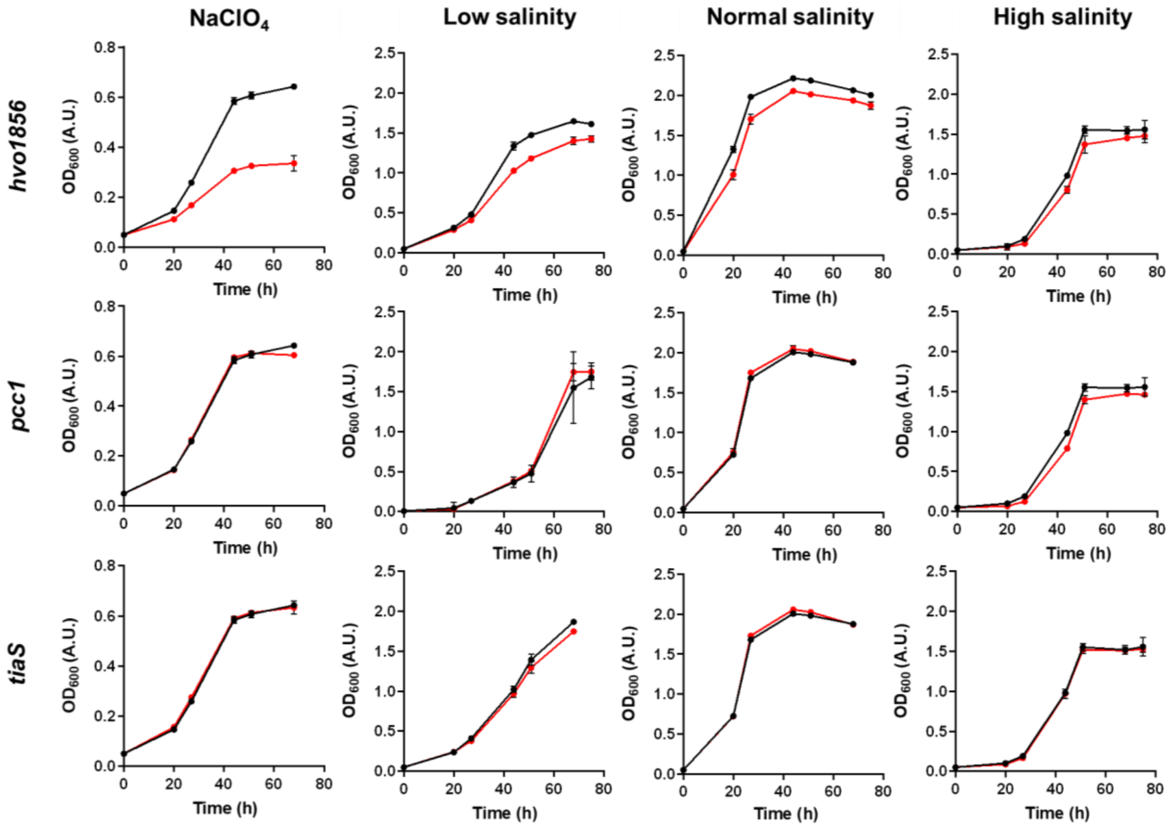

Figure S2. Growth curves of *H. volcanii* overexpressing certain genes involved in tRNA modifications. *H. volcanii* overexpressing *hvo1856*, *pcc1* and *tiaS* genes (red lines) and control strain (empty plasmid; black line) in the presence of 350 mM $NaClO_4$, and low (11% sea water), normal (18% sea water) and high (30% sea water) salinity. *trm1* gene could not be correctly cloned in the pAJ plasmid. Data represent the mean ± S.D. (n=3).

Table S1. Primers used in this work. Underlined bases indicate restriction sites.

Gene	Primer forward (5'-3')	Primer reverse (5'-3')
arcS	GGTGGTCATATGGAGAGCATC CAGAGGTCGTC	GGTGGTCTCGAGCCATGGACCT GTGCATAGAA
hvo1856	GGTGGTCATATGCGTAGCTCTG GACGTGGTC	GGTGGTAAGCTTGCCGGTAAGA CGTGGAACT
pcc1	AATCGTCATATGCTAAAACACC CGCGGTCTC	GGTGGTAAGCTTAAGAAGTCGC CGTCGAGTAG
tiaS	GGTACTCATATGCCATAATCGA CCCGAAAGG	GGTGGTAAGCTTGTGCTGGTGG TTCAGGACT
trm1	GGTGGTCATATGGTACAACCG CATCGCACAAT	GGTGGTAAGCTTGACGCCGTAG GTCGGTTAC

Data S1. Expression level of *H. volcanii* genes under tested conditions. The effect of three different treatments was analysed: YPC medium (control), 350 mM $NaClO_4$ and 350 mM NaCl (saline control). Comparison between NaCl and control was used to study the effects of salinity (S), $NaClO_4$ and control for the effects of perchlorate and salinity (PS), and $NaClO_4$ and saline control for the effects of perchlorate anion (P). Genes considered as DEGs showed a fold-changes larger than 1.8 and a *P*-adj value lower than 0.1. FC: fold-change.
